# Genomic insights on carotenoid synthesis by extremely halophilic archaea *Haloarcula* *rubripromontorii* BS2, *Haloferax* *lucentense* BBK2 and *Halogeometricum* *borinquense* E3 isolated from the solar salterns of India

**DOI:** 10.1038/s41598-024-70149-4

**Published:** 2024-08-30

**Authors:** Devika. N. Nagar, Kabilan Mani, Judith M. Braganca

**Affiliations:** 1 Dept of Biological Sciences, Birla Institute of Technology and Science, Pilani, K K Birla Goa Campus, NH 17B Zuarinagar, Goa, 403 726 India; 2 Center for Molecular Medicine & Therapeutics, PSG Institute of Medical Sciences and Research, Coimbatore, India

**Keywords:** Carotenoids, Halophilic archaea, Genomic diversity, WGS, Salinity, Computational biology and bioinformatics, Microbiology

## Abstract

Haloarchaeal cultures were isolated from solar salterns of Goa and Tamil Nadu and designated as BS2, BBK2 and E3. These isolates grew with a characteristic bright orange to pink pigmentation and were capable of growing in media containing upto 25% (w/vol) NaCl. Whole genome sequencing (WGS) of the three haloarchaeal strains BS2, BBK2 and E3 indicated an assembled genomic size of 4.1 Mb, 3.8 Mb and 4 Mb with G + C content of 61.8, 65.6 and 59.8% respectively. Phylogenetic analysis based on the 16S rRNA gene sequence revealed that the archaeal isolates belong to *Haloarcula*, *Haloferax* and *Halogeometricum* genera. *Haloarcula rubripromontorii* BS2  was predicted to have 4292 genes with 4242 CDS regions, 46 tRNAs, 6 rRNAs and 3 misc_RNAs. In case of *Haloferax* *lucentense*  BBK2, 3840 genes with 3780 CDS regions were detected along with 52 tRNAs, 5 rRNAs and 3 misc_RNAs. *Halogeometricum* *borinquense*  E3 contained 4101 genes, 4043 CDS regions, 52 tRNAs, 4 rRNAs, and 2 misc_RNAs. The functional annotation and curation of the haloarchaeal genome, revealed C50 carotenoid biosynthetic genes like phytoene desaturase/carotenoid 3′ -4′ desaturase (*crtI*), lycopene elongase (*ubiA*/*lyeJ*) and carotenoid biosynthesis membrane protein (*cruF*) in the three isolates. Whereas *crtD* (C-3′,4′ desaturase)*, crtY* (lycopene cyclase) and *brp*/*blh* (β-carotene dioxygenase) genes were identified only in BS2.

## Introduction

Halophilic microorganisms are prevalent in high salt environments and are found in all the three domains of life; Bacteria, Archaea and Eukarya^1^. They are usually categorised as extremely halophilic (> 15% NaCl), moderately halophilic (3–15% NaCl), slightly halophilic (1–3% NaCl), halotolerant and non-halophiles (< 1% NaCl)^2^. Solar salterns often exhibit vibrant colors owing to the abundance of pigmented microorganisms. These include *Dunaliella*, which is rich in β-carotene, bacteria such as *Salinibacter ruber*, known for producing the carotenoid salinixanthin, and halophilic archaea primarily producing C50 carotenoids^3^. Haloarchaea exhibit a vibrant red–orange hue attributed to carotenoid pigments such as bacterioruberin (BR) and its derivatives. Nevertheless, non- pigmented species such as *Natrialba asiatica* and *Halorhabdus tiamatea* have been reported^4^. Bacterioruberin is an isoprenoid derived carotenoid and is membrane bound thus yielding red, pink, orange pigmented colonies^5,6^.

Carotenoids are generally involved as accessory pigments in photosynthesis, as antioxidants, membrane stabilizers and in photoprotection. Depending on the number of carbons in their carotene backbone they are classified as C30, C40 and C50^7^. They are synthesized from the universal C5 precursor molecule isopentenyl pyrophosphate (IPP) and its isomer dimethylallyl pyrophosphate (DMAPP), which is derived from the terpenoid biosynthetic pathways with mevalonic acid being an intermediate, therefore known as the mevalonate pathway (MVA)^8,9^. Among different carotenoids in nature, C50 carotenoids mainly bacterioruberin and its intermediates bisanhydrobacterioruberin (BABR), monoanhydrobacterioruberin (MABR), and 2-isopentenyl-3,4-dehydrorhodopin (IDR) are mostly harboured in halophilic archaea^10–12^. C50 membrane bound carotenoids might help the archaeal cells adapt in hypersaline conditions, provide stability under high osmotic stress, protect from UV radiations, DNA damage and other oxidative stresses^13–16^. The antioxidant property of carotenoids attributes to its chemical structure, specifically the number of conjugated double bonds, carbon chain length and pigment concentration ^17,18^. Bacterioruberin (C50 carotenoid) contains 13 pairs of conjugated double bonds compared to only 9 pairs in well-known antioxidant β-carotene (C30 carotenoid), therefore bacterioruberin exhibits better radical scavenging properties than β-carotene^8,19,20^. Interestingly haloarchaea have the ability to synthesise both C40 and C50 carotenoids, of which most abundant are the C50 carotenoids^10,13,16^. There are only a few genomic and pathway related studies for C50 carotenoid biosynthesis in haloarchaea such as *Halorubrum ruber* sp.^21^; *Haloarcula japonica* ^7^; *Halobellus ruber*^22^ etc. In this study whole genome sequence analysis was used for the genomic characterization and identification of the hypothetical pathway involved in the C50 carotenoid biosynthesis in the three haloarchaeal strains BS2, BBK2 and E3 isolated in our laboratory from salterns of India^23,24^. This work gives insights into the carotenoid biosynthesis by different species of halophilic archaea.

## Methods and materials

### Archaeal strains and genomic DNA extraction

Haloarchaeal strains, BS2 and BBK2 were isolated from salterns of Ribandar (15°30′ N, 73°51′ E), Goa, India ^23^ whereas strain E3 was isolated from solar salterns of Marakkanam (12°14′ N, 79°55′ E) in Tamil Nadu, India^24^. The cultures were maintained on extremely halophilic medium (EHM), containing (g•L-1) NaCl 250; MgSO_4_ anhydrous 9.67; CaCl_2_•2H_2_O 0.36; NaHCO_3_ 0.06; NaBr 0.23; Peptone 5.0; Yeast extract 10.0; FeCl_3_•6H_2_O trace, pH 7.5^24^. For DNA isolation the three cultures were grown in EHM broth at 37 ℃ at 110 rpm till the cells reached late exponential phase (after 7 days). The cells were harvested and the genomic DNA was extracted using Wizard® Genomic DNA Purification Kit (Promega, USA), according to the manufacturer’s instruction. The DNA quality was quantified using Qubit® 2.0 and agarose gel electrophoresis.

### Genome sequencing/ Illumina sequencing

The paired-end sequence library was prepared using Illumina Nextera XT DNA Library Preparation Kit. The whole genome sequencing (WGS) was performed using 151 bp paired-end sequencing protocol on Illumina platform using HiSeq4000 sequencer according to the manufacturer’s instructions. Briefly, for the library preparation DNA was enzymatically fragmented by the enzyme mix provide by the Nextra XT DNA kit followed by adapter ligation of the fragments to generate sequencing libraries. Indexing adapters were ligated to the ends of the DNA fragments, preparing them for hybridization onto a flow cell. The ligated products were purified using AMPure XP beads supplied in the kit. The size-selected product was PCR amplified as described in the kit protocol. The amplified library was analysed in HiSeq4000 sequencer using High Sensitivity (HS) DNA chip as per manufacturer's instructions. After obtaining the Qubit concentration for the library, it was loaded onto HiSeq for cluster generation and sequencing. The kit reagents were used in binding of samples to complementary adapter oligos on paired-end flow cell. The adapters were designed to allow selective cleavage of the forward strands after re-synthesis of the reverse strand during sequencing. The copied reverse strand was then used to sequence from the opposite end of the fragment.

### Genomic assembly annotation and bioinformatic analysis

Quality assessment of the raw fastq reads of the samples was performed using FastQC v.0.11.9 tool at default parameters^25^. The raw fastq reads were pre-processed using Fastp v.0.20.1^26^. The high-quality processed data was de novo assembled using SKESA v.2.4.0^27^. Assembly completeness and the rate of contamination was assessed using CheckM^28^. The assemblies were assessed by Quast v. 5.2.0 tool^29^. Further the generated assemblies were annotated for gene prediction using Prokka v. 1.14.6^30^. The protein sequence file generate by Prokka was used for the upcoming bioinformatic analysis and the second round of annotation was done by RAST v. 2.0^31,32^. Numerical distribution of the annotated genes was represented into different subsystems obtained in both the pie chart and list format. Protein sequence (.faa) generated by Prokka was subjected to PANNZER2^33^ and BlastKOALA v.3.0^34^ for functional characterization and annotation of unknown  proteins. Circular genome map of the draft assembly was generated using PROKSEE^35^. The Prokka annotated proteins were further aligned against the blast databases using DIAMOND blastp v.2.0.6^36^sequence aligner and subjected to Blast2GO v.6.0.3^37^. The Gene ontology (GO) terms were subjected to WEGO v. 2.0^38^ online server for enrichment of GO terms. The biosynthetic gene clusters (BGCs) respective to carotenoid biosynthetic gene cluster were predicted by antiSMASH v.7.0.^39^. Presence of possible CRISPR sequences and associated Cas protein sequences were determined by CRISPERCasFinder web tool^40^. PlasmidFinder and pMLST was employed for identification of plasmid sequences, if any in the genome sequence^41^. PHASTER web tool^42,43^ was used to find any prophage regions in the sequenced genomes.

### Phylogeny analysis and genome comparison

16S rRNA gene based taxonomy assessment for the assembled genome of B2, BBK2 and E3 was performed using the 16S rRNA gene based ID service of EzBioCloud^44^. The evolutionary history based on the 16S rRNA sequences of the assembled genomes and 28 closest 16S rRNA sequences was inferred using the Neighbor-Joining method^45^. The evolutionary distances were computed using the Maximum Likelihood method and Tamura-Nei model^46^. Phylogenetic tree was constructed to show the evolutionary position of these isolates by employing MEGA X^47^. Average amino acid identity (AAI) and in silico DNA-DNA hybridization (*is*DDH) were used to calculate genomic relatedness and delineate the microbial species. ANI values were computed as per the method described by Yoon et al^48^ and *is*DDH values were computed from Genome-to-Genome Distance Calculator^49^. Heatmap was generated with OrthoANI value calculated from the OAT software^50^. Genomic comparison analysis was performed by circular genome map of the draft assembly generated using PROKSEE tool^35^.

## Results and discussion

The haloarchaeal strains BS2 and BBK2, isolated from solar salterns located in Ribandar, Goa, India^23^ along with strain E3, which was isolated from solar salterns in Marakkanam, Tamil Nadu, India^24^, were used in the study. These isolates had characteristic orange pink pigments and were further studied using genomic sequencing. The quality of genomic DNA extracted from haloarchaeal strains BS2, BBK2 and E3 was quantified using Qubit® 2.0 and agarose gel electrophoresis (Supplementary Fig. 1).

### Sequencing, assembly, phylogenetic analysis, and general features of the genomes

The genomic DNA libraries of the three haloarchaeal isolates were prepared by Nextera XT DNA Library Preparation Kit and the genome sequence was determined using illumina HiSeq sequencer. The Illumina-compatible sequencing library for the sample had 14,954,776 reads with an average fragment size of 853 bp for strain BS2, 14,468,630 reads with an average fragment size of 875 bp for strain BBK2 and 15,163,526 reads with an average fragment size of 788 bp for strain E3^25^ and the raw fastq reads were pre-processed using Fastp v.0.20.1^26^. The genome wide de novo assembly was done using SKESA v.2.4.0^27^. The assembled genomic size of *Haloarcula rubripromontorii* BS2 was 4.1 Mb with N50 value of 508,287 bp, while for *Haloferax lucentense* BBK2 the genomic size was 3.8 Mb with N50 value of 211,896 bp and *Halogeometricum borinquense* E3 had a genome size of 4 Mb with N50 value of 240303 bp. The G + C content of the three haloarchaeal strains was 61.8, 65.6 and 59.8% for BS2, BBK2 and E3 respectively. It’s not surprising to learn that these haloarchaeal isolates have a higher GC content in the genomic DNA as previous reports on halophilic archaea have indicated the same^51^.

**Fig. 1:**
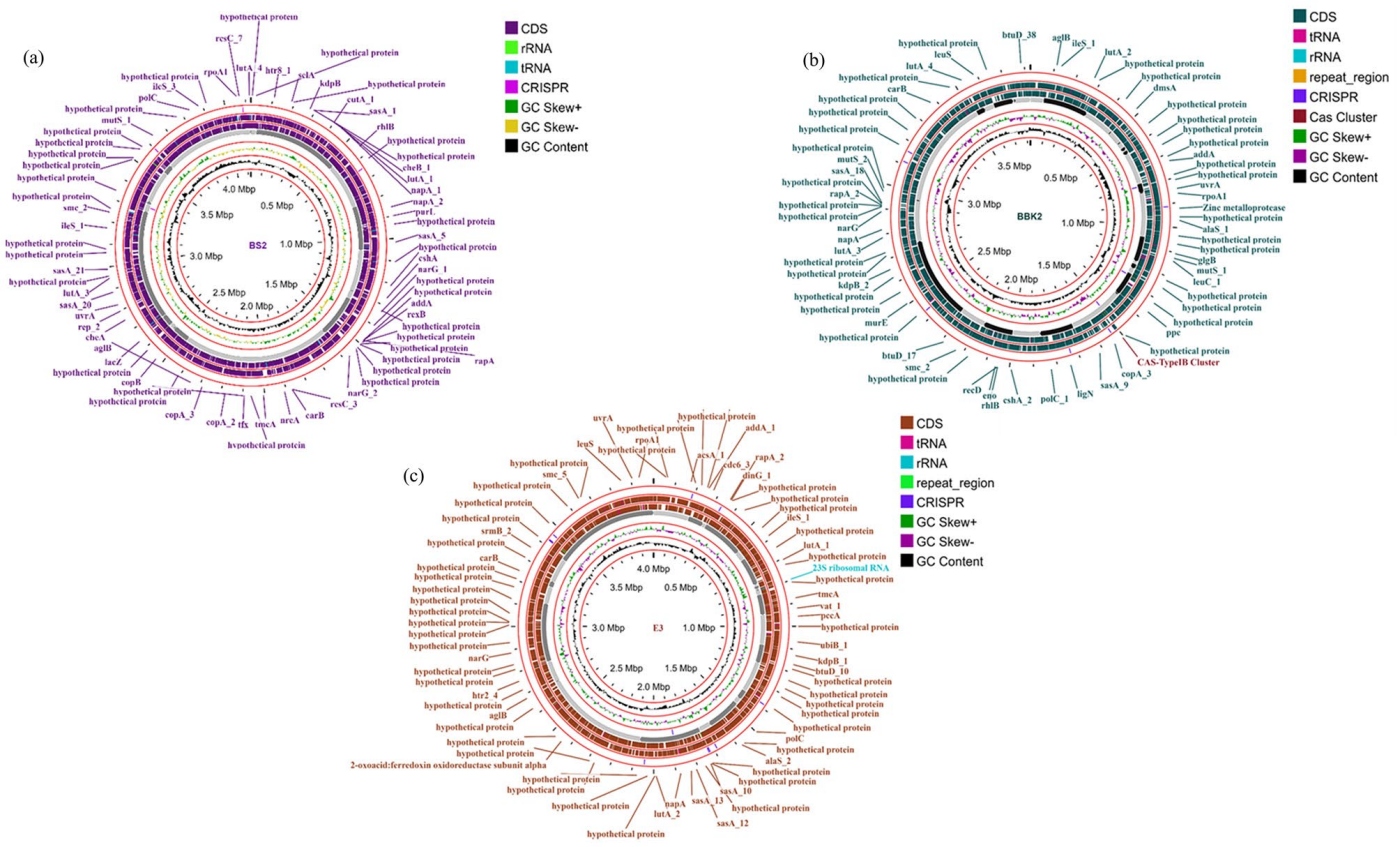
Circular Genome Plot of *Haloarcula rubripromontorii* BS2 (**a**); *Haloferax lucentense* BBK2 (**b**) and *Halogeometricum borinquense* E3 (**c**). A graphical circular map of the genome was performed with Proksee tool. The outermost ring 1 (Forward strand) and ring 2 (Reverse strand), show the protein coding regions. The 3rd Ring in Grey, shows the contigs. Ring 4 shows the GC skew and ring 5 shows the GC content.

**Fig. 2:**
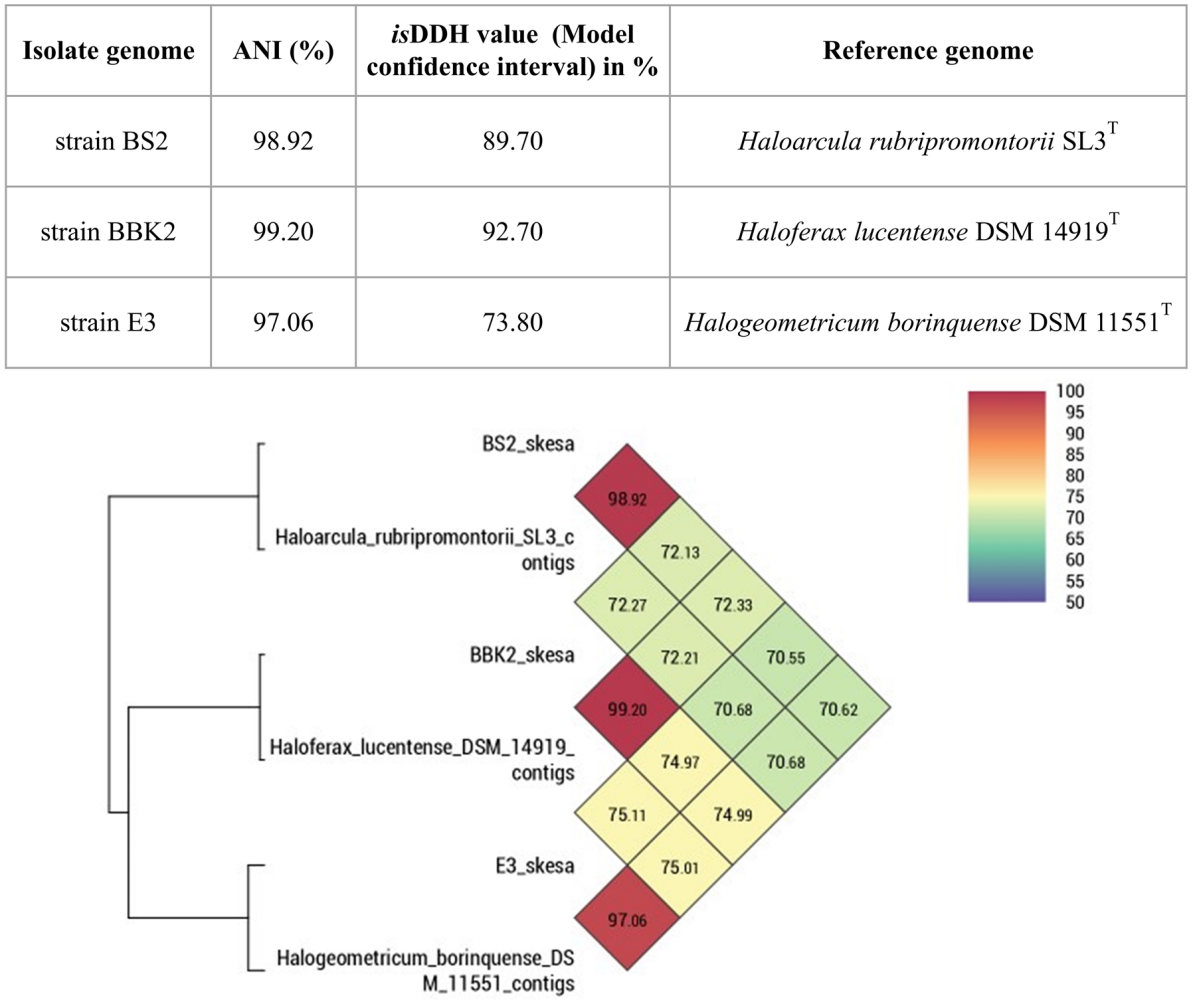
Genomic DNA relatedness based on OrthoANI values (also as heatmap) and in-silico DDH (*is*DDH) values between strains BS2; BBK2 and E3 and closely related species of genera *Haloarcula*; *Haloferax* and *Halogeometricum* respectively.

Similar to bacterial cells archaea generally contain extrachromosomal plasmids responsible for various functions. However, the genomic DNA extracted from all the three strains in this study lacked plasmid sequences. It is known that CRISPR arrays, along with their Cas proteins, provide bacteria and archaea with a form of adaptive immunity, defending them against exogenous phages or plasmids. CRISPRCasFinder is a tool that enables the detection of both CRISPR arrays and Cas proteins^40^. In BS2, BBK2 and E3 strains 3, 7 and 6 possible CRISPR repeat sequences were identified. BS2 strain contained 2 possible prophage regions, whereas both BBK2 and E3 strains had 1 possible prophage region (Table. 1). A graphical representation of the coding sequence (CDS) regions, RNAs, CRISPR sequences, and GC content for the three isolates constructed using Proksee tool is shown in Fig. 1. Taxonomic analysis for 16S  rRNA gene sequences obtained from whole genome sequencing of the three strains BS2, BBK2 and E3 via 16S based ID service of EzBioCloud revealed similarity percentage of 97.75, 99.66 and 100% similarity to the closely related phenotype *Haloarcula rubripromontorii, Haloferax lucentense* and *Halogeometricum borinquense* respectively. Phylogenetic tree (Supplementary Fig. 2) based on 16S rRNA gene sequences also confirmed with the above closest clustered species. In addition, OrthoANI values computed for BS2, BBK2 and E3 strains, when compared with closely related species based on the 16S  rRNA gene sequences were 98.92, 99.20 and 97.06% and *is*DDH values computed for the same were 89.7, 92.7 and 73.8% respectively (Fig. 2).

**Table 1:** General characterization and annotation statistics of *Haloarcula* sp. BS2, *Haloferax* sp. BBK2 and *Halogeometricum* sp. E3.

Isolate	Strain BS2	Strain BBK2	Strain E3
Size (bp)	4125613	3825065	4000899
GC content	61.8%	65.69%	59.88%
Number of contigs	22	37	28
Number of large contigs (> 1000 bp)	19	32	27
Bases in large contigs (> 1000 bp)	4123385	3821983	4000252
Largest contig length (bp)	869213	573511	604532
N50	508287	211896	240303
L50	4	6	5
Number of subsystems	190	197	197
CDS	4242	3780	4043
Genes	4297	3840	4101
RNAs			
tRNAs	46	52	52
rRNAs	6	5	4
Misc_RNAs	3	3	2
Repeat regions	–	3	4
CRISPR repeats	3	7	6
Plasmid sequences	0	0	0
Phage sequences	2	1	1

The assembly statistics were calculated by QUAST version 5.0.2. The tools used for functional genome annotation and analysis: PROKKA; RAST; CRISPRCasFinder; Plasmid Finder and PHASTER.

### Genomic prediction and genome annotation

**Fig. 3:**
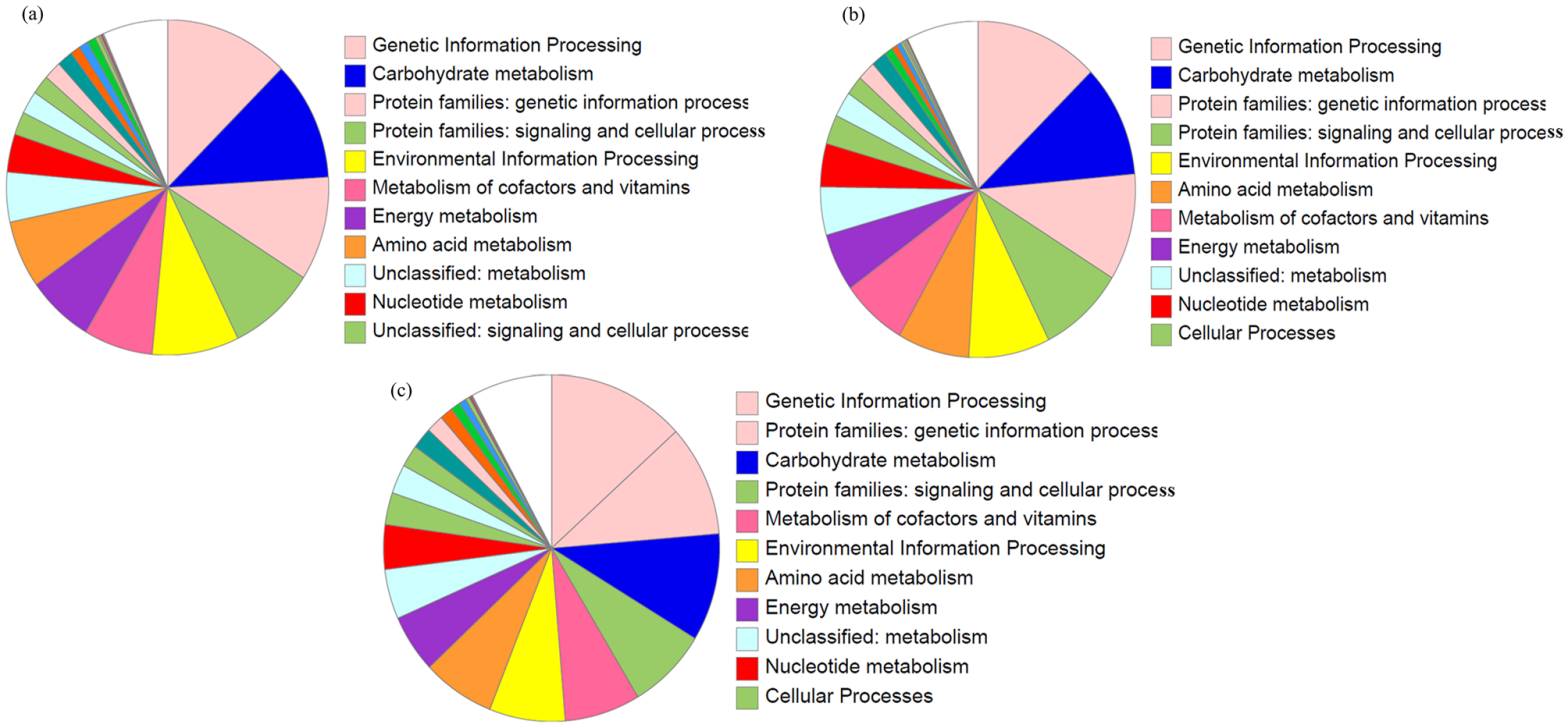
Graphical view of genomic annotated functional categories by BlastKOALA Kyoto Encyclopedia of Genes and Genomes (KEGG), predicted in strains BS2 (**a**); BBK2 (**b**) and E3 (**c**).

Genomic annotation analysis using Prokka tool  predicted *Haloarcula* sp. BS2 has 22 contigs, 4292 genes with 4242 CDS regions, 46 tRNAs, 6 rRNAs and 3 misc_RNAs. In case of *Haloferax* sp. BBK2, 37 contigs, 3840 genes with 3780 CDS regions were detected along with 52 tRNAs, 5 rRNAs, 3 misc_RNAs and 3 repeat regions. *Halogeometricum* sp. E3 contained 28 contigs with 4101 genes, 4043 CDS regions, 52 tRNAs, 4 rRNAs, 2 misc_RNAS and 4 repeat regions (Table. 1). The protein sequences were further aligned against databases using DIAMOND blastp v.2.0.6 for Blastp alignment and annotation, which provided gene lengths, e-values, GO IDs, enzyme code and names. Annotated protein sequences were subjected to Blast2GO v.6.0.3^37^ and then GO terms were subjected to the WEGO v.2.0^38^ for enrichment and plotting Gene ontology (GO) annotation data (Supplementary Fig. 3). GO statistics using WEGO represented 3,015, 2,919 and 2,881 total annotated genes for BS2, BBK2 and E3, which are divided into three classes: Biological process (1743, 1750 and 1694 respectively of annotated genes); Molecular function (2318, 2236 and 2186 respectively of annotated genes) and Cellular components (1501, 1442 and 1458 respectively of annotated genes) (Supplementary Table. 1). Functional annotation by PANNZER2 tool provided information about the GO annotations and descriptive prediction of annotated genes specific for enzymes involved in pigment production and diversity among the cultures. Annotated data was also represented by performing BlastKOALA resulting in 38.3, 41.1 and 37.2% annotated output for BS2, BBK2 and E3 respectively and it assigned KEGG ontology for the annotated genes according to the functional characterization of individual genes (Fig. 3). RASTtk SEED data output of BS2 revealed 4396 number of coding sequences with 625 (15%) featured in subsystems and 3771 (85%) were not in any subsystems. Similarly in the case of BBK2 number of coding sequences were found to be 4259 with 634 (15%) featured in subsystems and 3625 (85%) were not in any subsystems, whereas in case of E3 number of coding sequences were 4246 with 612 (15%) featured in subsystems and 3625 (85%) were not in any subsystems (Supplementary Fig. 4). For these cultures the number of coding sequences featured in a subsystem encoded for 612 (97.9%) as non-hypothetical proteins while 13 (2%) as hypothetical proteins for BS2; 625 (98.6%) as non-hypothetical while 9 (1.4%) as hypothetical for BBK2 and 610 (99.7%) as non-hypothetical whereas 11 (1.8%) as hypothetical for E3 strain. Likewise, the number of coding sequences not featured in any subsystem encoded 1021 (27%) as non-hypothetical proteins while 2750 (72.9%) as hypothetical in the case of BS2; whereas for BBK2 930 (25.7%) were non-hypothetical proteins whereas 2694 (74.3%) were hypothetical and lastly for E3 941 (25.9%) were non-hypothetical proteins and 2684 (74%) were hypothetical. antiSMASH results showed (Table. 2) that all three strains possess a terpene cluster.

**Table 2:** Distribution of BGCs respective to terpene and carotenoid biosynthesis gene clusters of strains BS2; BBK2 and E3 detected by antiSMASH 2.0 server.

Isolate	Type	Compound	Span (nt)	Clusterblast similarity (%)	Taxon of similar gene clusters
BS2	Terpene	Carotenoid	192,338–193,393	100	*Haloarcula* *vallismortis* DSM 3756^T^
			206,669–207,640	100	*Haloarcula* *vallismortis* DSM 3756^T^
BBK2	Terpene	Carotenoid	13,919–14,938,	95	*Haloferax* *volcanii* DS2^T^
			151,031–151,987	90	*Haloferax* *volcanii* DS2^T^
E3	Terpene	Carotenoid	65,657–66,673	78	*Halogeometricum* *borinquense *DSM 11551^T^
			194,175–195,245,	100	*Halogeometricum* *borinquense* DSM 11551^T^

### Bacterioruberin biosynthesis

Genomic annotations of all three cultures predicted C50 carotenoid synthesis (through terpenoid biosynthesis) via mevalonic acid (MVA) pathway. The gene geranylgeranyl diphosphate synthase type I (*idsA1*) responsible for the synthesis of precursor molecule geranylgeranyl pyrophosphate (GGPP) necessary for the carotenoid biosynthesis by the MVA pathway was annotated in all the three isolates BS2, BBK2 and E3. The C50 carotenoid synthesis pathway includes a 15-cis-phytoene synthase (*crtB*), lycopene elongase (*ubiA*/*lyeJ*) and carotenoid biosynthesis membrane protein (*cruF*) genes which were found in all three isolates thus confirming the C50 carotenoid synthesis. An important gene *crtI* here is been identified and annotated as phytoene desaturase and carotenoid 3′-4′ desaturase in all the three isolates, whereas *crtD* annotated as C-3′,4′ desaturase (CrtD), *crtY* annotated as lycopene beta cyclase (CrtY) and *brp*/*blh* annotated as β-carotene dioxygenase (Brp) were identified only in *Haloarcula* sp. BS2. It has been reported that *Haloarcula japonica* seems to have two types desaturation reactions involving CrtI enzyme for the first reaction converting phytoene to lycopene and CrtD in the second reaction forming double bonds at C-3,4 and C-3′,4′ of the lycopene derivatives. Further both these genes have been reported to show homology for CrtD and CrtI enzymes, thus indicating their identical functionalities^7^. According to PANNZER functional annotation, the *crtI* annotated as phytoene desaturase/carotenoid 3′, 4′ desaturase and *crtD* annotated as C-3′,4′ desaturase, thus, it could be hypothesised that *Haloarcula* sp. BS2 has both the desaturase enzyme (CrtD and CrtI) required in bacterioruberin C50 carotenoid biosynthesis. Whereas, in the other two isolates *Haloferax* sp. BBK2 and *Halogeometricum* sp. E3 only *crtI* gene was identified for the two desaturase enzyme activities required in the pathway. Other than *crtD* (C-3′,4′ desaturase)*, crtY* and *brp*/*blh* genes encoding for lycopene cyclase and β-carotene dioxygenase respectively were also not identified in BBK2 and E3 isolates. Gene *crtY* is involved in β-carotene biosynthesis from lycopene and followed by retinal and bacteriorhodopsin biosynthesis. Therefore, in this study, we report that *crtD*, *crtY* and *brp/bhl* genes were found to be specific only for *Haloarcula* sp. BS2 (Table 3). Available studies report that only a few species of *Haloarcula*^7^ and *Halobacterium* are able to synthesise bacteriorhodopsin, which has also shown to inhibit bacterioruberin synthesis^52^. *Haloferax* and *Halogeometricum* species are not known to synthesize bacteriorhodopsin and therefore accumulate only bacterioruberin. Thus, our data indicates that there is a diversity within the haloarchaeal species with regards to bacterioruberin synthesis and could be one of the reasons for the variation in shades of red, orange and pink colonies among these species^53^.

**Table 3:** Functionally annotated genes of terpenoid backbone and carotenoid biosynthesis pathways for strains.

Gene	Enzyme name	NCBI RefSeq accession			KEGG no
		strain BS2	strain BBK2	strain E3	
Terpenoid backbone pathway					
idi	Isopentenyl-diphosphate delta-isomerase	WP_004593679.1	WP_004042477.1	WP_163486965.1	K01823
idsA	Geranylgeranyl diphosphate synthase	WP_004593534.1 and WP_004594002.1	WP_004044620.1 and WP_004042941.1	WP_163486744.1 and WP_163485183.1	K13787
Carotenoid biosynthesis pathway					
crtB	15-cis-phytoene synthase	WP_004591065.1	WP_004042512.1	WP_163486845.1	K02291
lyeJ/ubiA	Lycopene elongase/Prenyltransferase	M0L7V9.1	WP_144858537.1	QIB75025.1	K20616
cruF	Bisanhydrobacterioruberin hydratase	WP_004593286.1	WP_004042518.1	WP_163486846.1	K08977
crtI	Phytoene/Carotenoid 3,4-desaturase	WP_004593284.1 and WP_004591214.1	WP_004042523.1	WP_163486848.1	K20611
crtD	C-3′,4′ desaturase	WP_004591113.1	–	–	
crtY	lycopene beta cyclase	WP_004591067.1	–	–	K22502
bhl/brp	beta-carotene 15,15′-dioxygenase	WP_004591069.1 and EMA33692.1	–	–	K21817

BS2; BBK2 and E3 with KEGG number, involved in the different reported terpenoid backbone and carotenoid biosynthesis pathways.

**Fig. 4:**
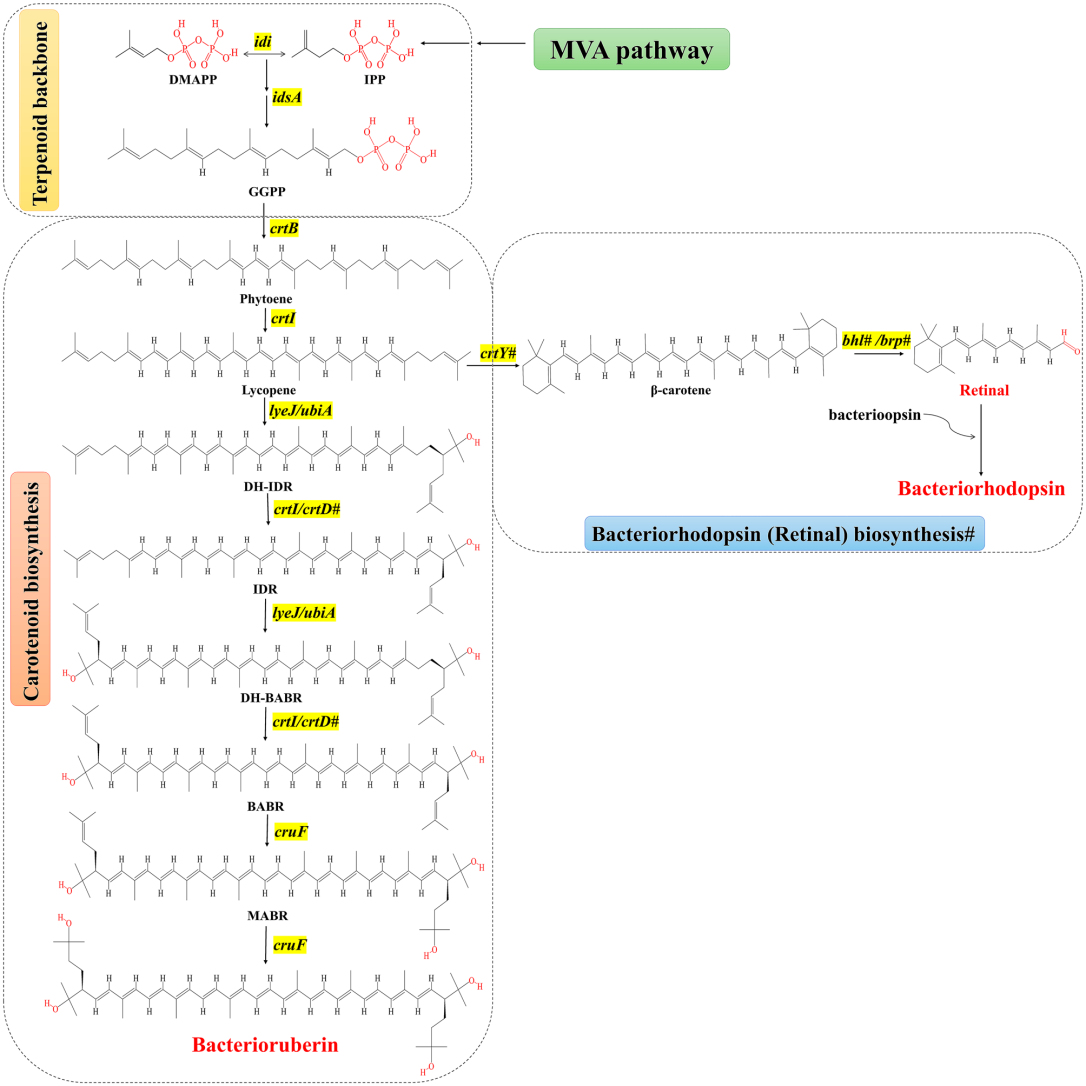
Predicted C50 (bacterioruberin) carotenoid biosynthesis pathway in *Haloarcula rubripromontorii* BS2, *Haloferax lucentense* BBK2 and *Halogeometricum* *borinquense* E3 based on genome annotation. Gene annotated for retinal biosynthesis was only found in BS2 strains. # indicated for the biosynthesis and gene annotation specific to BS2 strain.

The genes thus involved in carotenogenesis in *Haloarcula* *rubripromontorii* BS2, *Haloferax* *lucentense* BBK2 and *Halogeometricum* *borinquense* E3, have been depicted through a biosynthetic pathway in Fig. 4. The heat map representing these annotated genes is shown in Supplementary Fig. 5. The antiSMASH output predicted the three strains BS2, BBK2 and E3 to have two biosynthetic gene clusters (BGCs) responsible for the secondary metabolites of terpene. ClusterBlast identified that two BGCs showed similarities in gene cluster of *Haloarcula vallismortis* DSM 3756^ T^ for BS2; *Haloferax volcanii* DS2^T^ for BBK2 and *Halogeometricum borinquense* DSM 11551^ T^ for E3. According to KnownClusterBlast, these three isolates share no similarity with any known pathways. The phytoene synthase gene (*crtB*) was represented by the core biosynthetic gene of terpene BGCs in all the three strains. Nucleotide locations of both the BGCs associated with carotenoid biosynthesis of each of the strains were identified and represented in the Table. 2.

Haloarchaeal isolates express brilliant red - orange pigment attributed to bacterioruberin. These unique C50 pigments provide membrane stability, UV protection, ROS scavenging, etc., to the halophilic archaea^13–16^. Haloarchaea inhabit extreme hypersaline econiches and are known to produce various potential novel bioactive compounds. C50 carotenoids structurally exhibit better antioxidative properties due to their extended conjugated double bonds and the inclusion of at least one hydroxyl group^17,18^. Consequently, this uncommon class of carotenoids is appealing for various biological and industrial applications. Our sequencing data revealed the genes related to C50 carotenoid synthesis and diversity within the biosynthetic pathway among the haloarchaeal cultures. In-depth genomic analysis of the C50 carotenoid pathway in haloarchaeal species is not much elucidated. This study thus focused on giving insights into the genomic characterization of different species of halophilic archaea and the hypothetical pathway involved in the C50 carotenoid biosynthesis in them. This will open new avenues by promoting whole genome guided bioprospecting approach to carotenogenesis and other biotechnological applications of haloarchaeal species.

## Supplementary Information

Supplementary Information.

## Data Availability

Whole Genome sequencing projects have been deposited at DDBJ/ENA/GenBank under the accession numbers JAWJXX000000000, JAWLKZ000000000, JAXCMF000000000 for *Haloarcula* sp. BS2, *Haloferax* sp. BBK2 and *Halogeometricum* sp. E3 respectively. The respective raw sequence reads have also been deposited in the Sequence read archive (SRA) database under Bioproject ID PRJNA1028943, PRJNA1027579 and PRJNA1029887.

## References

[1] Oren, A. Halophilic microbial communities and their environments. *Curr. Opin. Biotechnol.* **33**, 119–124. 10.1016/j.copbio.2015.02.005 (2015). 25727188 10.1016/j.copbio.2015.02.005

[2] Ventosa, A. & Nieto, J. J. Biotechnological applications and potentialities of halophilic microorganisms. *World J. Microbiol. Biotechnol.* **11**, 85–94. 10.1007/BF00339138 (1995). 24414413 10.1007/BF00339138

[3] de Lourdes Moreno, M., Sánchez-Porro, C., García, M. T. & Mellado, E. Carotenoids’ production from halophilic bacteria. In *Microbial Carotenoids from Bacteria and Microalgae: Methods and Protocols* (ed. Barredo, J.-L.) 207–217 (Humana, 2012). 10.1007/978-1-61779-879-5_12 22623305

[4] Salgaonkar, B. B. & Rodrigues, R. a study on the halophilic archaeal diversity from the food grade iodised crystal salt from a saltern of India. *Microbiology (N Y)* **88**, 709–719. 10.1134/S002626171906016X (2019).

[5] Oren, A. & Rodríguez-Valera, F. The contribution of halophilic Bacteria to the red coloration of saltern crystallizer ponds (1). *FEMS Microbiol Ecol* **36**, 123–130. 10.1111/j.1574-6941.2001.tb00832.x (2001). 11451516 10.1111/j.1574-6941.2001.tb00832.x

[6] Amoozegar, M. A., Siroosi, M., Atashgahi, S., Smidt, H. & Ventosa, A. Systematics of haloarchaea and biotechnological potential of their hydrolytic enzymes. *Microbiology (N Y)* **163**, 623–645. 10.1099/mic.0.000463 (2017). 10.1099/mic.0.000463 28548036

[7] Yang, Y. *et al.* Complete biosynthetic pathway of the C_50_ carotenoid bacterioruberin from lycopene in the extremely halophilic archaeon *Haloarcula japonica*. *J. Bacteriol.* **197**, 1614–1623. 10.1128/JB.02523-14 (2015). 25712483 10.1128/JB.02523-14 PMC4403650

[8] Yatsunami, R. *et al.* Identification of carotenoids from the extremely halophilic archaeon *Haloarcula japonica*. *Front. Microbiol.* **5**, 100. 10.3389/fmicb.2014.00100 (2014). 24672517 10.3389/fmicb.2014.00100 PMC3956123

[9] Heider, S. A. E., Wolf, N., Hofemeier, A., Peters-Wendisch, P. & Wendisch, V. F. Optimization of the IPP precursor supply for the production of lycopene, decaprenoxanthin and astaxanthin by *Corynebacterium**glutamicum*. *Front. Bioeng. Biotechnol.* **2**, 28. 10.3389/fbioe.2014.00028 (2014). 25191655 10.3389/fbioe.2014.00028 PMC4138558

[10] Rodrigo-Baños, M., Garbayo, I., Vílchez, C., Bonete, M. J. & Martínez-Espinosa, R. M. Carotenoids from haloarchaea and their potential in biotechnology. *Mar. Drugs* **13**, 5508–5532. 10.3390/md13095508 (2015). 26308012 10.3390/md13095508 PMC4584337

[11] Montero-Lobato, Z. *et al.* Optimization of growth and carotenoid production by haloferax mediterranei using response surface methodology. *Mar. Drugs* **16**, 372. 10.3390/md16100372 (2018). 30304770 10.3390/md16100372 PMC6213265

[12] Kelly, M. & Jensen, S. L. Bacterial carotenoids. XXVI. C50-carotenoids. 2 Bacterioruberin. *Acta Chem. Scand.* **21**, 2578–2580. 10.3891/acta.chem.scand.21-2578 (1967). 5585680 10.3891/acta.chem.scand.21-2578

[13] Giani, M., Garbayo, I., Vílchez, C. & Martínez-Espinosa, R. M. Haloarchaeal carotenoids: Healthy novel compounds from extreme environments. *Mar. Drugs* **17**, 524. 10.3390/md17090524 (2019). 31500208 10.3390/md17090524 PMC6780574

[14] Fang, C.-J., Ku, K.-L., Lee, M.-H. & Su, N.-W. Influence of nutritive factors on C50 carotenoids production by Haloferax mediterranei ATCC 33500 with two-stage cultivation. *Bioresour. Technol.* **101**, 6487–6493. 10.1016/j.biortech.2010.03.044 (2010). 20362434 10.1016/j.biortech.2010.03.044

[15] Chen, C. W., Hsu, S. H., Lin, M. T. & Hsu, Y. H. Mass production of C50 carotenoids by Haloferax mediterranei in using extruded rice bran and starch under optimal conductivity of brined medium. *Bioprocess Biosyst. Eng.* **38**, 2361–2367. 10.1007/s00449-015-1471-y (2015). 26373421 10.1007/s00449-015-1471-y

[16] Grivard, A. *et al.* Archaea carotenoids: Natural pigments with unexplored innovative potential. *Mar. Drugs* **20**, 524. 10.3390/md20080524 (2022). 36005527 10.3390/md20080524 PMC9410494

[17] Miller, N. J., Sampson, J., Candeias, L. P., Bramley, P. M. & Rice-Evans, C. A. Antioxidant activities of carotenes and xanthophylls. *FEBS Lett.* **384**, 240–242. 10.1016/0014-5793(96)00323-7 (1996). 8617362 10.1016/0014-5793(96)00323-7

[18] Albrecht, M., Takaichi, S., Steiger, S., Wang, Z. Y. & Sandmann, G. Novel hydroxycarotenoids with improved antioxidative properties produced by gene combination in *Escherichia coli*. *Nat. Biotechnol.* **18**, 843–846. 10.1038/78443 (2000). 10932152 10.1038/78443

[19] Saito, T., Miyabe, Y., Ide, H. & Yamamoto, O. Hydroxyl radical scavenging ability of bacterioruberin. *Radiat. Phys. Chem.* **50**, 267–269. 10.1016/S0969-806X(97)00036-4 (1997).

[20] Stahl, W. & Sies, H. (2005). Bioactivity and protective effects of natural carotenoids. *Biochimica et Biophysica Acta (BBA) - Molecular Basis of Disease.* **1740**, 101–107. 10.1016/j.bbadis.2004.12.006 10.1016/j.bbadis.2004.12.006 15949675

[21] Hwang, C. Y., Cho, E. S., Rhee, W. J., Kim, E. & Seo, M. J. Genomic and physiological analysis of C50 carotenoid-producing novel Halorubrum ruber sp. nov. *J. Microbiol.* **60**, 1007–1020. 10.1007/s12275-022-2173-1 (2022). 36029458 10.1007/s12275-022-2173-1

[22] Hwang, C. Y., Cho, E. S., Yoon, D. J. & Seo, M. J. Halobellus ruber sp. Nov., a deep red-pigmented extremely halophilic archaeon isolated from a Korean solar saltern. *Antonie Van Leeuwenhoek* **114**, 997–1011. 10.1007/s10482-021-01571-1 (2021). 33864546 10.1007/s10482-021-01571-1

[23] Mani, K., Salgaonkar, B. B. & Braganca, J. M. Culturable halophilic archaea at the initial and crystallization stages of salt production in a natural solar saltern of Goa. *India. Aquat Biosyst..* **8**, 15. 10.1186/2046-9063-8-15 (2012). 22747590 10.1186/2046-9063-8-15 PMC3444409

[24] Salgaonkar, B. B. & Bragança, J. M. Biosynthesis of poly(3-hydroxybutyrate-co-3-hydroxyvalerate) by Halogeometricum borinquense strain E3. *Int. J. Biol. Macromol.* **78**, 339–346. 10.1016/j.ijbiomac.2015.04.016 (2015). 25895957 10.1016/j.ijbiomac.2015.04.016

[25] Andrews, S. FastQC: a quality control tool for high throughput sequence data. www.bioinformatics.babraham.ac.uk/projects/fastqc/ (2010).

[26] Chen, S., Zhou, Y., Chen, Y. & Gu, J. fastp: An ultra-fast all-in-one FASTQ preprocessor. *Bioinformatics* **34**, i884–i890. 10.1093/bioinformatics/bty560 (2018). 30423086 10.1093/bioinformatics/bty560 PMC6129281

[27] Souvorov, A., Agarwala, R. & Lipman, D. SKESA: Strategic k-mer extension for scrupulous assemblies. *Genome Biol.* **19**, 153. 10.1186/s13059-018-1540-z (2018). 30286803 10.1186/s13059-018-1540-z PMC6172800

[28] Parks, D. H., Imelfort, M., Skennerton, C. T., Hugenholtz, P. & Tyson, G. W. CheckM: Assessing the quality of microbial genomes recovered from isolates, single cells, and metagenomes. *Genome Res.* **25**, 1043–1055. 10.1101/gr.186072.114 (2015). 25977477 10.1101/gr.186072.114 PMC4484387

[29] Gurevich, A., Saveliev, V., Vyahhi, N. & Tesler, G. QUAST: Quality assessment tool for genome assemblies. *Bioinformatics* **29**, 1072–1075. 10.1093/bioinformatics/btt086 (2013). 23422339 10.1093/bioinformatics/btt086 PMC3624806

[30] Seemann, T. Prokka: Rapid prokaryotic genome annotation. *Bioinformatics* **30**, 2068–2069. 10.1093/bioinformatics/btu153 (2014). 24642063 10.1093/bioinformatics/btu153

[31] Aziz, R. K. *et al.* The RAST server: Rapid annotations using subsystems technology. *BMC Genomics* **9**, 75. 10.1186/1471-2164-9-75 (2008). 18261238 10.1186/1471-2164-9-75 PMC2265698

[32] Overbeek, R. *et al.* The SEED and the rapid annotation of microbial genomes using subsystems technology (RAST). *Nucl. Acids Res.* **42**, D206–D214. 10.1093/nar/gkt1226 (2014). 24293654 10.1093/nar/gkt1226 PMC3965101

[33] Törönen, P., Medlar, A. & Holm, L. PANNZER2: A rapid functional annotation web server. *Nucl. Acids Res.* **46**, W84–W88. 10.1093/nar/gky350 (2018). 29741643 10.1093/nar/gky350 PMC6031051

[34] Kanehisa, M., Sato, Y. & Morishima, K. BlastKOALA and GhostKOALA: KEGG tools for functional characterization of genome and metagenome sequences. *J. Mol. Biol.* **428**, 726–731. 10.1016/j.jmb.2015.11.006 (2016). 26585406 10.1016/j.jmb.2015.11.006

[35] Grant, J. R. *et al.* Proksee: In-depth characterization and visualization of bacterial genomes. *Nucl. Acids Res.* **51**, W484–W492. 10.1093/nar/gkad326 (2023). 37140037 10.1093/nar/gkad326 PMC10320063

[36] Buchfink, B., Reuter, K. & Drost, H. G. Sensitive protein alignments at tree-of-life scale using DIAMOND. *Nat. Methods* **18**, 366–368. 10.1038/s41592-021-01101-x (2021). 33828273 10.1038/s41592-021-01101-x PMC8026399

[37] Conesa, A. & Götz, S. Blast2GO: A comprehensive suite for functional analysis in plant genomics. *Int. J. Plant Genomics* **2008**, 619832. 10.1155/2008/619832 (2008). 18483572 10.1155/2008/619832 PMC2375974

[38] Ye, J. *et al.* WEGO 2.0: A web tool for analyzing and plotting GO annotations, 2018 update. *NucL. Acids Res.* **46**, W71–W75. 10.1093/nar/gky400 (2018). 29788377 10.1093/nar/gky400 PMC6030983

[39] Blin, K. *et al.* antiSMASH 7.0: New and improved predictions for detection, regulation, chemical structures and visualisation. *Nucl. Acids Res* **51**, W46–W50. 10.1093/nar/gkad344 (2023). 37140036 10.1093/nar/gkad344 PMC10320115

[40] Couvin, D. *et al.* CRISPRCasFinder, an update of CRISRFinder, includes a portable version, enhanced performance and integrates search for Cas proteins. *Nucl. Acids Res.* **46**, W246–W251. 10.1093/nar/gky425 (2018). 29790974 10.1093/nar/gky425 PMC6030898

[41] Carattoli, A. *et al.* In silico detection and typing of plasmids using plasmidfinder and plasmid multilocus sequence typing. *Antimicrob. Agents Chemother.* **58**, 3895–3903. 10.1128/AAC.02412-14 (2014). 24777092 10.1128/AAC.02412-14 PMC4068535

[42] Arndt, D. *et al.* PHASTER: A better, faster version of the PHAST phage search tool. *Nucl. Acids Res.* **44**, W16–W21. 10.1093/nar/gkw387 (2016). 27141966 10.1093/nar/gkw387 PMC4987931

[43] Zhou, Y., Liang, Y., Lynch, K. H., Dennis, J. J. & Wishart, D. S. PHAST: A fast phage search tool. *Nucl. Acids Res.* **39**, W347–W352. 10.1093/nar/gkr485 (2011). 21672955 10.1093/nar/gkr485 PMC3125810

[44] Yoon, S. H. *et al.* Introducing EzBioCloud: A taxonomically united database of 16S rRNA gene sequences and whole-genome assemblies. *Int. J. Syst. Evol. Microbiol.* **67**, 1613–1617. 10.1099/ijsem.0.001755 (2017). 28005526 10.1099/ijsem.0.001755 PMC5563544

[45] Saitou, N. & Nei, M. The neighbor-joining method: A new method for reconstructing phylogenetic trees. *Mol. Biol. Evol.* **4**, 406–425. 10.1093/oxfordjournals.molbev.a040454 (1987). 3447015 10.1093/oxfordjournals.molbev.a040454

[46] Tamura, K. & Nei, M. Estimation of the number of nucleotide substitutions in the control region of mitochondrial DNA in humans and chimpanzees. *Mol. Biol. Evol.* **10**, 512–526. 10.1093/oxfordjournals.molbev.a040023 (1993). 8336541 10.1093/oxfordjournals.molbev.a040023

[47] Kumar, S., Stecher, G., Li, M., Knyaz, C. & Tamura, K. MEGA X: Molecular evolutionary genetics analysis across computing platforms. *Mol. Bio.l Evol.* **35**, 1547–1549. 10.1093/molbev/msy096 (2018). 10.1093/molbev/msy096 PMC5967553 29722887

[48] Yoon, S. H., Ha, S. M., Lim, J., Kwon, S. & Chun, J. A large-scale evaluation of algorithms to calculate average nucleotide identity. *Antonie Van Leeuwenhoek* **110**, 1281–1286. 10.1007/s10482-017-0844-4 (2017). 28204908 10.1007/s10482-017-0844-4

[49] Meier-Kolthoff, J. P., Carbasse, J. S., Peinado-Olarte, R. L. & Göker, M. TYGS and LPSN: A database tandem for fast and reliable genome-based classification and nomenclature of prokaryotes. *Nucl. Acids Res.* **50**, D801–D807. 10.1093/nar/gkab902 (2022). 34634793 10.1093/nar/gkab902 PMC8728197

[50] Lee, I., Ouk Kim, Y., Park, S. C. & Chun, J. OrthoANI: An improved algorithm and software for calculating average nucleotide identity. *Int. J. Syst. Evol. Microbiol.* **66**, 1100–1103. 10.1099/ijsem.0.000760 (2016). 26585518 10.1099/ijsem.0.000760

[51] Lynch, E. A. *et al.* Sequencing of seven haloarchaeal genomes reveals patterns of genomic flux. *PLoS ONE* **7**, e41389. 10.1371/journal.pone.0041389 (2012). 22848480 10.1371/journal.pone.0041389 PMC3404096

[52] Dummer, A. M. *et al.* Bacterioopsin-mediated regulation of bacterioruberin biosynthesis in *Halobacterium**salinarum*. *J. Bacteriol.* **193**, 5658–5667. 10.1128/JB.05376-11 (2011). 21840984 10.1128/JB.05376-11 PMC3187228

[53] Giani, M., Miralles-Robledillo, J. M., Peiró, G., Pire, C. & Martínez-Espinosa, R. M. Deciphering pathways for carotenogenesis in haloarchaea. *Molecules* **25**, 1197. 10.3390/molecules25051197 (2020). 32155882 10.3390/molecules25051197 PMC7179442

