# Author Correction: Genomic insights on carotenoid synthesis by extremely halophilic archaea *Haloarcula rubripromontorii* BS2, *Haloferax lucentense* BBK2 and *Halogeometricum borinquense* E3 isolated from the solar salterns of India

**DOI:** 10.1038/s41598-024-74079-z

**Published:** 2024-10-10

**Authors:** Devika. N. Nagar, Kabilan Mani, Judith M. Braganca

**Affiliations:** 1 Dept of Biological Sciences, Birla Institute of Technology and Science, Pilani, K K Birla Goa Campus, NH 17B, Zuarinagar, Goa 403 726 India; 2 Center for Molecular Medicine & Therapeutics, PSG Institute of Medical Sciences and Research, Coimbatore, India

Correction to: *Scientific Reports* 10.1038/s41598-024-70149-4, published online 30 August 2024

The original version of this Article contained errors in the Results and Discussion, under the subheading ‘Bacterioruberin biosynthesis’,

“Whereas, in the other two isolates *Haloferax* sp. BBK2 *Halofera *sp. And *Halogeometricum* sp. E3 *Halogeometricum* sp. only *crtI* gene was identified for the two desaturase enzyme activities required in the pathway.”

now reads:

“Whereas, in the other two isolates *Haloferax* sp. BBK2 and *Halogeometricum* sp. E3 only *crtI* gene was identified for the two desaturase enzyme activities required in the pathway.”

The original Article has been corrected.

