# Supplementary material for: Genomic insights on carotenoid synthesis by extremely halophilic archaea Haloarcula rubripromontorii BS2, Haloferax lucentense BBK2 and Halogeometricum borinquense E3 isolated from the solar salterns of India
Source: Sci Rep. 2024 Aug 30;14:20214. doi: 10.1038/s41598-024-70149-4 (PMC11364659; doi:10.1038/s41598-024-70149-4)
Supplement: Supplementary file 1 — Supplementary Information. [file 41598_2024_70149_MOESM1_ESM.docx]

# **Genomic insights on carotenoid synthesis by extremely halophilic archaea *Haloarcula* *rubripromontorii* BS2, *Haloferax lucentense* BBK2 and *Halogeometricum* *borinquense* E3 isolated from the solar salterns of India**

Devika. N. Nagar ^1^, Kabilan Mani ^2^, Judith M Braganca ^1^*

^1^ Dept of Biological Sciences, Birla Institute of Technology and Science, Pilani, K K Birla Goa campus, NH 17B Zuarinagar, Goa 403 726, India

^2^ Center for Molecular Medicine & Therapeutics, PSG Institute of Medical Sciences and Research, Coimbatore, India

* Corresponding authors: Author Three ([judith@goa.bits-pilani.ac.in](mailto:judith@goa.bits-pilani.ac.in))

**
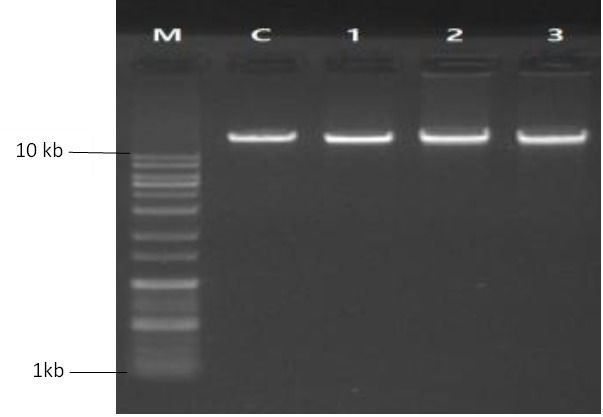
Supplementary Fig. 1**: Agarose gel electrophoresis of genomic DNA extracted from haloarchaeal strains E3, BBK2 and BS2 (lane 1- 3), M and C indicate 1 kb ladder and lambda DNA as control.


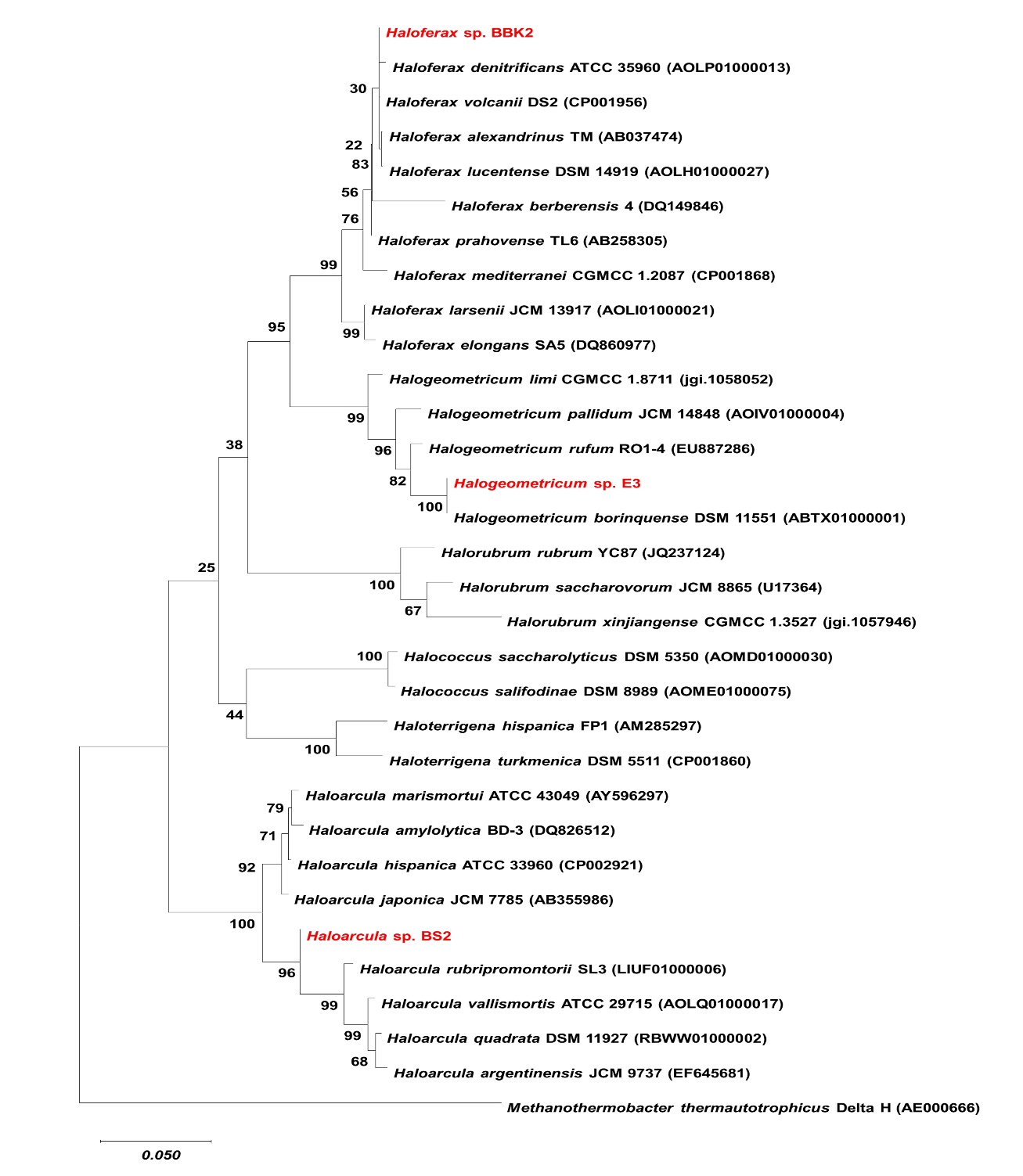
**Supplementary Fig. 2**: Maximum likelihood (ML) phylogenetic Tree based on the 16S rRNA gene sequences. Phylogenetic tree was constructed with closely related species. The evolutionary history was inferred by using the Maximum Likelihood method and Tamura-Nei model. The percentage of trees in which the associated taxa clustered together in the bootstrap test (1000 replicates) is shown next to the branches. Initial tree(s) for the heuristic search were obtained automatically by applying Neighbor-Join and BioNJ algorithms to a matrix of pairwise distances estimated using the Tamura-Nei model, and then selecting the topology with superior log likelihood value. Bar, 0.050 accumulated changes per nucleotide, respectively. Evolutionary analyses were conducted in MEGA X.


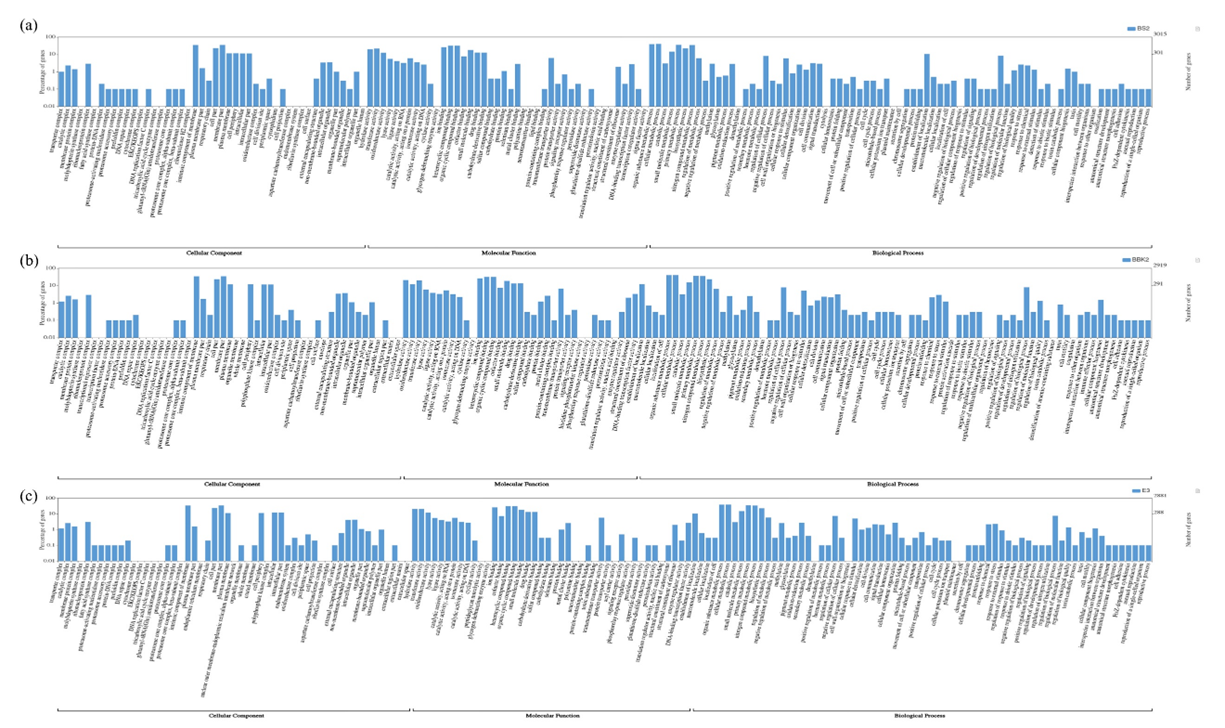


**Fig. 3**: Gene Ontology (GO) Bar plot of the strains BS2 (a); BBK2 (b) and E3 (c).


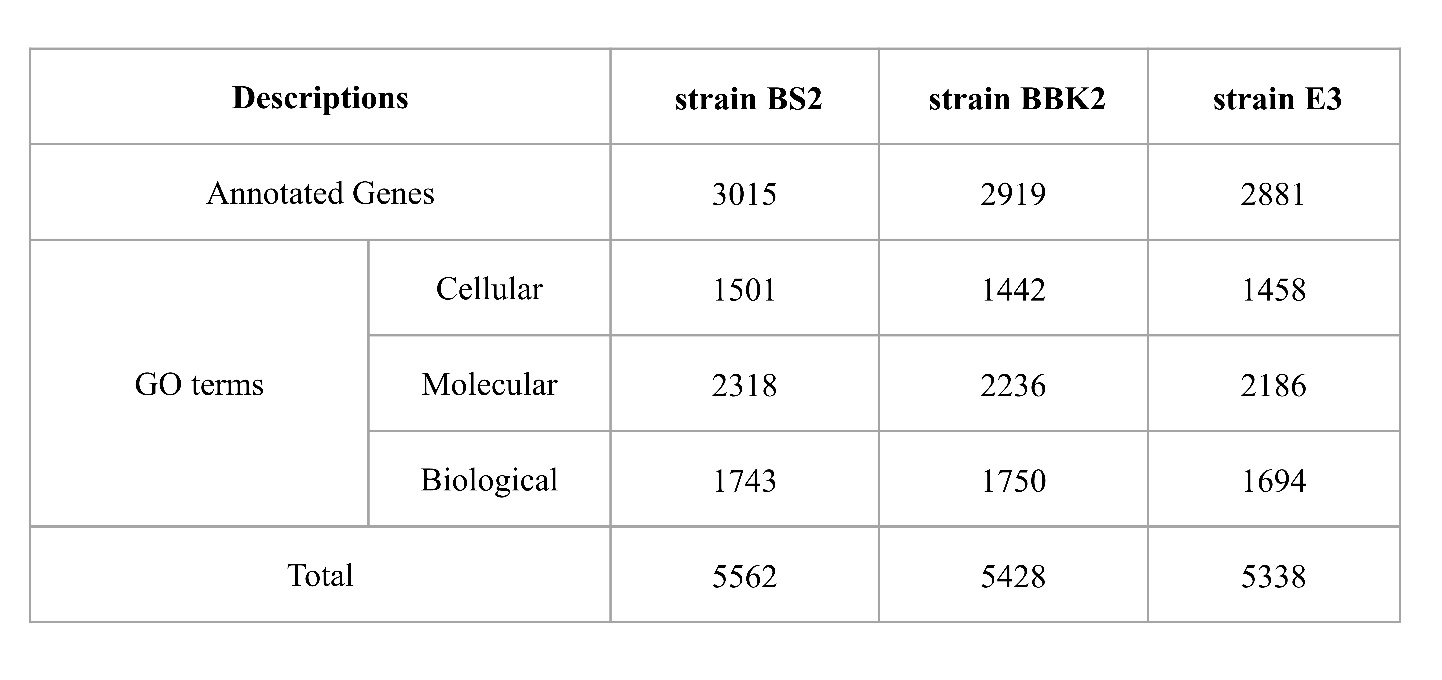
**Supplementary Table. 1:** Gene Ontology (GO) Statistics using WEGO representing GO annotated genes.


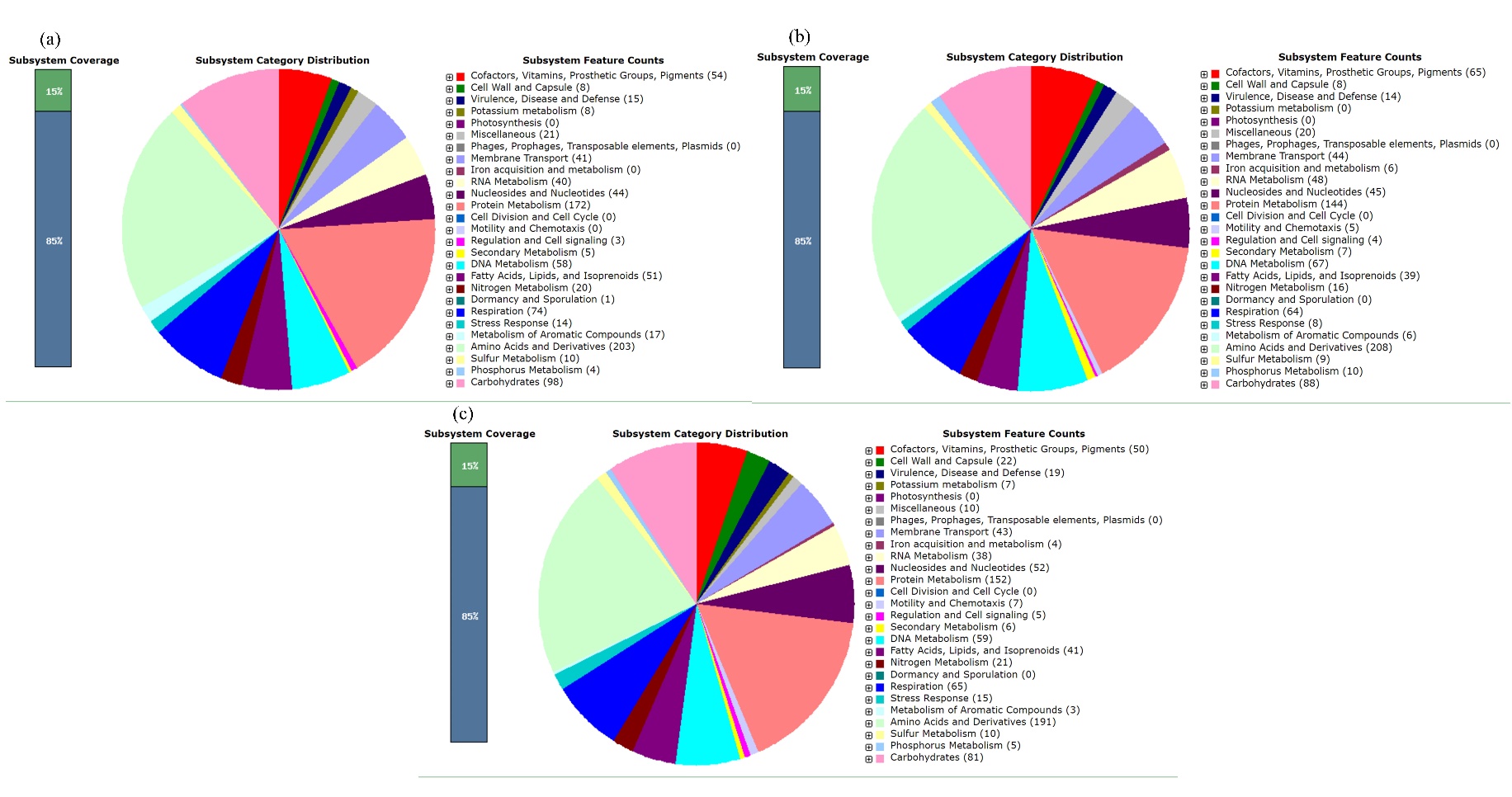


**Supplementary Fig. 4:** Clusters of Orthologous Groups (COGs) of protein function classification via RAST annotation of strains BS2 (a); BBK2 (b) and E3 (c). The subsystem coverage in percentage at left; each part of the pie graph indicates different functions and proportions of genes and the numbers in brackets shows the counts of genes with specific functions.


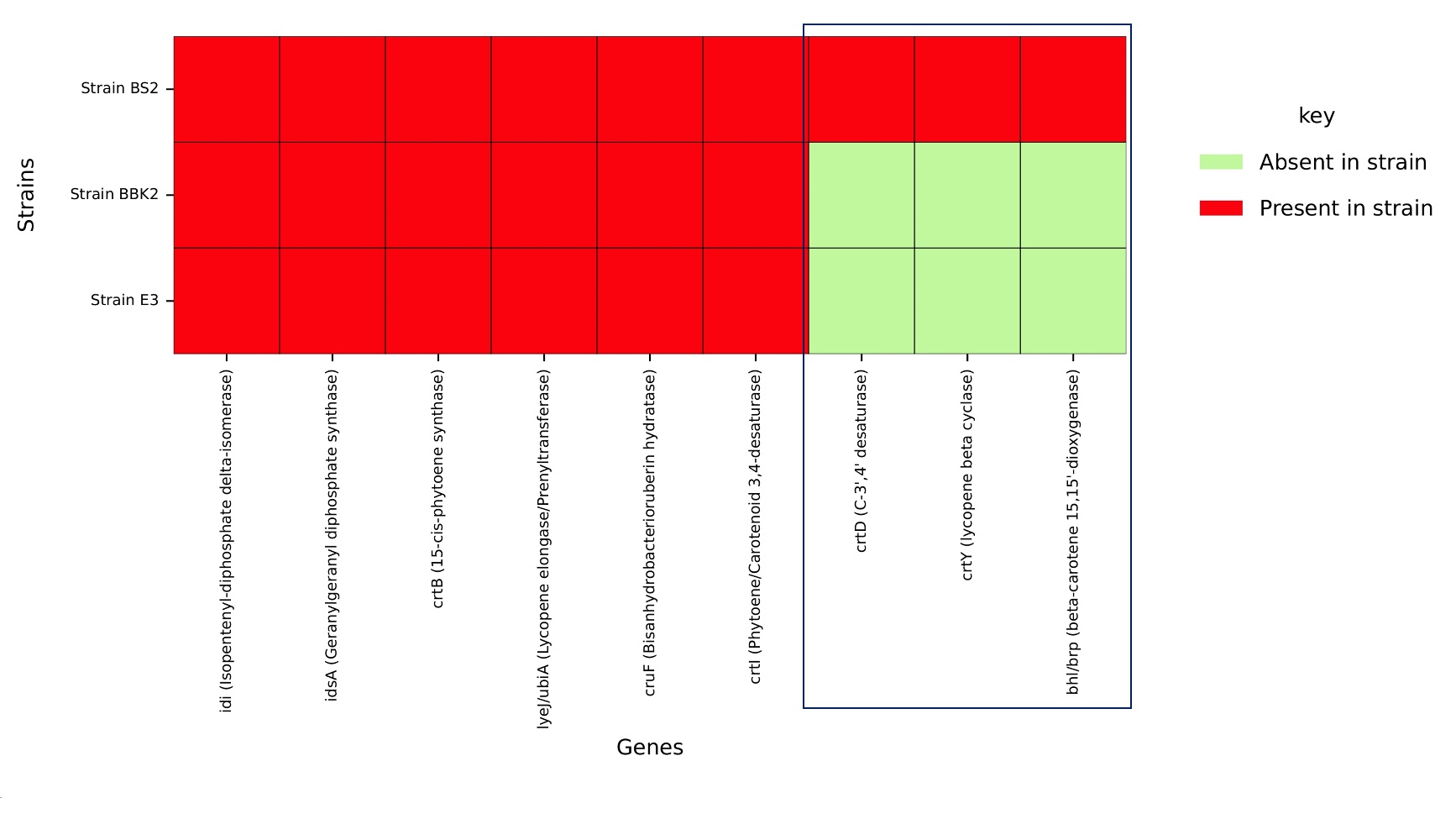


**Supplementary Fig. 5:** Heat map representing presence/absence matrix of genes for the enzymes involved in the carotenoid biosynthesis in strains BS2; BBK2 and E3.
